# Meta-analysis of the association between three microRNA polymorphisms and breast cancer susceptibility

**DOI:** 10.18632/oncotarget.18516

**Published:** 2017-06-16

**Authors:** Kun Mu, Zi-Zheng Wu, Jin-Pu Yu, Wei Guo, Nan Wu, Li-Juan Wei, Huan Zhang, Jing Zhao, Jun-Tian Liu

**Affiliations:** ^1^ The Second Department of Breast Cancer, Tianjin Medical University Cancer Institute and Hospital, National Clinical Research Center for Cancer, Key Laboratory of Cancer Prevention and Therapy, Tianjin, Tianjin's Clinical Research Center for Cancer, Tianjin 300060, P.R. China; ^2^ Department of Breast Surgery, The First Hospital of Qinhuangdao, Qinhuangdao 066000, P.R. China; ^3^ Cancer Molecular Diagnostic Center, Tianjin Medical University Cancer Institute and Hospital, National Clinical Research Center for Cancer, Key Laboratory of Cancer Prevention and Therapy, Tianjin, Tianjin's Clinical Research Center for Cancer, Tianjin 300060, P.R. China; ^4^ Department of Orthopaedics, Tianjin Medical University General Hospital, Tianjin 300052, P.R. China

**Keywords:** breast cancer, meta-analysis, miRNA, polymorphisms

## Abstract

Single nucleotide polymorphisms (SNPs) in three microRNAs (miRNAs), rs2910164 in miR-146a, rs11614913 in miR-196a2, and rs3746444 in miR-499, have been associated with breast cancer (BC) susceptibility, but the evidence is conflicting. To obtain a more robust assessment of the association between these miRNA variants and BC risk, we carried out a meta-analysis through systematic literature retrieval from the PubMed and Embase databases. A total of 9 case-control studies on rs2910164, 12 on rs11614913, and 7 on rs3746444 were included. Pooled odds ratios and 95% confidence intervals were used to evaluate associations with BC risk. Overall analysis showed that rs2910164 was not associated with BC susceptibility in any genetic model, whereas rs11614913 was associated with a decreased risk in both the allelic contrast and recessive models, and rs3746444 imparted an increased risk in all genetic models. Stratified analyses showed that rs11614913 may decrease the risk of BC in the heterozygote model in Asians, and in all genetic models, except the heterozygote model, when the sample size is ≥ 500. Subgroup analysis indicated that rs3746444 was associated with increased risk of BC in Asians, but not Caucasians, at all sample sizes. This meta-analysis suggests that rs11614913 in miR-196a2 may decrease the risk of BC, while rs3746444 in miR-499 may increase it, especially in Asians when the sample size is large. We propose that rs11614913(C > T) and rs3746444 (A > G) may be useful biomarkers predictive of BC risk.

## INTRODUCTION

One of the most surprising advances in understanding the mechanisms of gene regulation in health and disease has been the discovery of microRNA (miRNA) [1].

MiRNAs are short (usually 21–23 nucleotides in length), evolutionarily conserved, noncoding RNA molecules that exert post-transcriptional regulation via binding to complementary sequences in the 3′-untranslated region (3′ UTR) or 5′-untranslated region (5′UTR) of target messenger RNAs (mRNAs) [2, 3]. Upon miRNA binding, the mRNA transcript is degraded or its translation inhibited [4]. Thus, miRNAs play a crucial role in gene expression, affecting many normal and abnormal cellular processes such as cell differentiation, proliferation, metabolism, apoptosis, and tumorigenesis [5, 6].

Breast cancer (BC) is the most common type of cancer affecting women worldwide. Although its etiology is multifactorial, its development and outcome are especially influenced by genetic factors. In this regard, several studies pointed out that alterations in miRNAs may contribute to the pathogenesis of BC [6, 7]. Since approximately 50% of miRNA genes are located in cancer-related chromosomal regions [8], in recent years their usefulness as biomarkers to evaluate cancer risk has been the subject of intense research.

Single nucleotide polymorphisms (SNPs) represent the most common form of genetic variation. When present in miRNA genes, SNPs may influence miRNAs’ properties by altering their expression, maturation, and/or function [9], and may thus increase the risk of cancer, or influence its progression [10, 11].

Among several common miRNA SNPs purportedly related with BC risk, the association of rs2910164 in miR-146a, rs11614913 in miR-196a2, and rs3746444 in miR-499 and BC risk remains inconclusive. For instance, Bansal et al. found that the heterozygous variant of rs2910164 in miR-146a is associated with reduced risk of BC [12], while a separate report indicated associations for rs11614913 in miR-196a2 and rs3746444 in miR-499 [13]. Nevertheless, some studies reported that these polymorphisms were not related to BC risk [14, 15]. Therefore, in order to evaluate the association of these three miRNA SNPs and BC susceptibility, we performed this meta-analysis by systematically summarizing published data.

## RESULTS

### Characteristics of the studies

**Figure 1:**
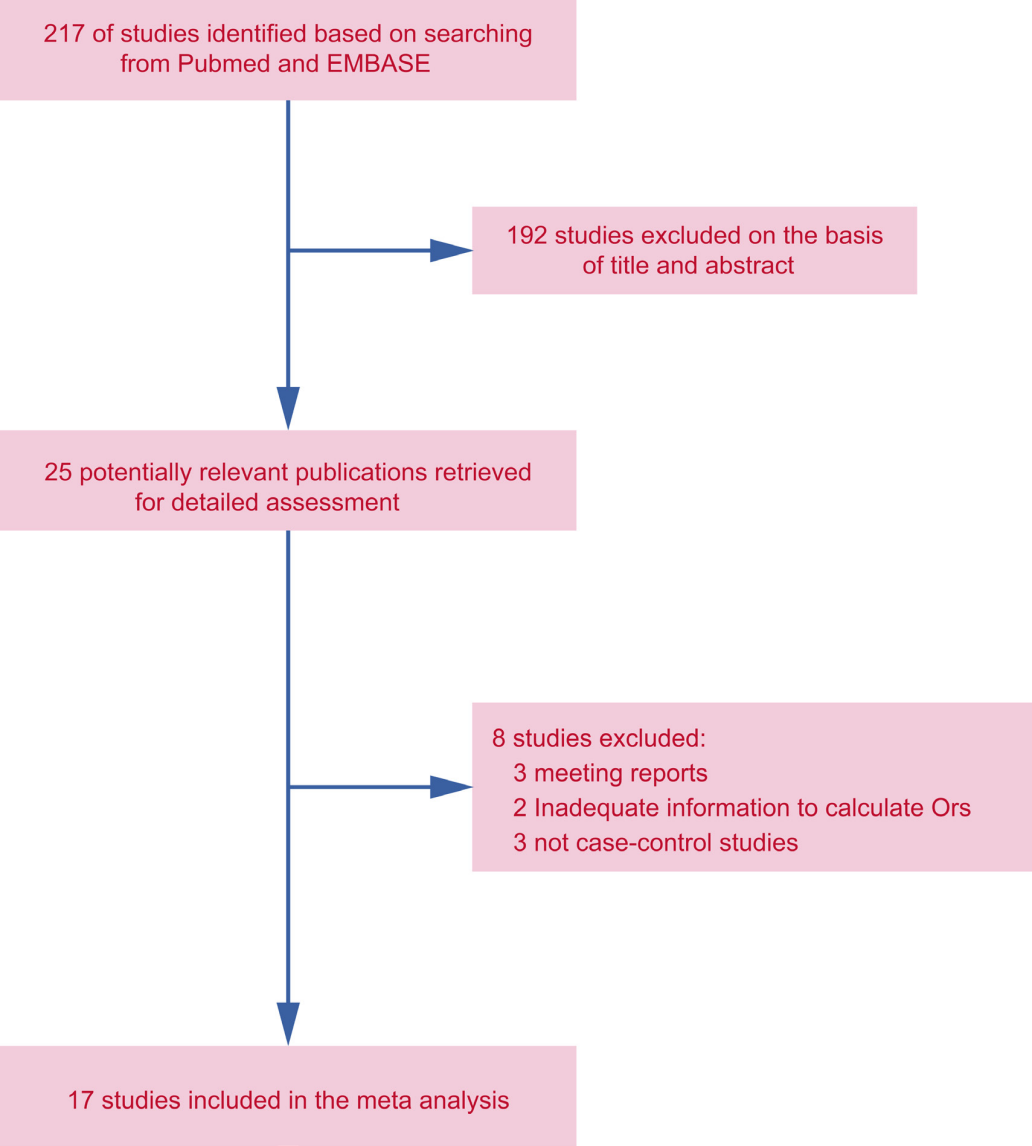
Study selection process

**Figure 2:**
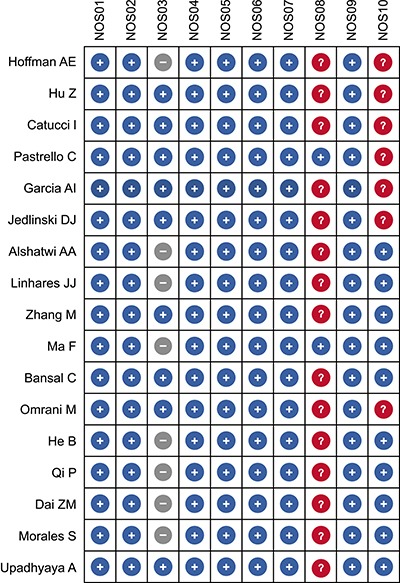
Quality assessment of included studies according to the Newcastle-Ottawa Scale (NOS) criteria)

Based on the search strategy, a total of 217 articles were chosen from PubMed and EMBASE databases. After screening the title and abstract, 192 articles uncorrelated with BC risk and miR-146a/-196a2/-499 SNPs were excluded. 25 articles were then evaluated in detail, and 8 articles were further excluded, among which 3 were meeting reports [16–18], 2 had inadequate information to calculate ORs [19, 20], and the other 3 were not case-control studies [21–23]. Finally, 17 eligible articles were included in our meta-analysis [12–15, 24–36] (Figure 1). The characteristics and the NOS quality assessment of the included studies are outlined in Table 1 and Figure 2. We categorized races as Asian (Chinese, Iranian, and Indian), Caucasian (Australian, Arab, Brazilian, French, Italian, German, and American), and Mixed (Chilean, and Non-Caucasian Brazilian) based on the original information from each study. In these articles, the distribution of genotypes in the controls were consistent with HWE in most of the studies, but some parts of the data in Qi’s, Omrani’s, Catucci’s, Ma’s, Bansal’s, Alshatwi’s, and Linhares’ studies didn't meet HWE. In Catucci's study, the subjects were from two countries, whereas in Linhares’ study the samples in the case and control groups belonged to Caucasian and non-Caucasian populations, so we treated them as independent studies. There were nine studies containing 4,441 cases and 3,899 controls for miR-146a rs2910164 [12–15, 24–26, 28, 36]; twelve studies involving 5,792 cases and 7,159 controls for miR-196a2 rs11614913 [13, 14, 27–35]; and seven studies including 4,019 cases and 4,683 controls for miR-499 rs3746444 [13, 14, 24, 28, 30, 35]. Genotype distributions in controls were in accord with HWE in all included studies. A variety of genotyping methods were applied including TaqMan, PCR-RFLP, MassARRAY, and HRM.

**Table 1:** The baseline characteristics for included studies

Study ID	Year	Country	Race	Genotyping	Source of Control	Case				Control				HWE (*P*)
**miR146a (rs2910164 G > C)**						**Total**	**GG**	**GC**	**CC**	**Total**	**GG**	**GC**	**CC**	
Upadhyaya	2016	Australia	Caucasian	HRM	PB	546	325	193	28	246	112	99	35	0.091
He	2015	China	Asian	MassARRAY	HB	450	75	242	133	450	72	225	153	0.478
Bansal	2014	India	Asian	PCR-RFLP	PB	121	82	35	4	164	84	72	8	0.130
Ma	2013	China	Asian	MassARRAY	HB	192	35	94	63	191	34	93	64	0.983
Alshatwi	2012	Arabia	Caucasian	TaqMan	HB	100	48	50	2	100	51	46	3	0.051
Garcia	2011	France	Caucasian	Taqman	PB	1130	676	388	66	596	352	220	24	0.150
Catucci	2010	Germany	Caucasian	Taqman	PB	805	451	304	50	904	536	318	50	0.753
Pastrello	2010	Italy	Caucasian	Taqman	PB	88	53	30	5	155	90	59	6	0.332
Hu	2009	China	Asian	PCR-RFLP	PB	1009	165	515	329	1093	180	551	362	0.221
**miR196a2 (rs11614913 C > T)**						**Total**	**CC**	**CT**	**TT**	**Total**	**CC**	**CT**	**TT**	
Morales	2016	Chile	Mix	TaqMan	HB	440	192	191	57	807	342	351	114	0.121
Dai	2016	China	Asian	MassARRAY	HB	560	197	265	98	583	155	284	144	0.540
Qi	2015	China	Asian	TaqMan	HB	321	34	119	168	290	17	88	185	0.141
He	2015	China	Asian	MassARRAY	HB	450	81	233	136	450	93	223	134	0.990
Omrani	2014	Iran	Asian	PCR- RFLP	PB	236	218	18	0	203	178	25	0	0.350
Zhang	2012	China	Asian	PCR- RFLP	PB	248	11	89	148	243	17	93	133	0.893
Linhares	2012	Brazil	Non-Caucasian	TaqMan	HB	63	11	29	23	114	33	51	30	0.264
Jedlinski	2011	Australia	Caucasian	PCR- RFLP	PB	187	68	86	33	171	58	82	31	0.830
Catucci	2010	Italy	Caucasian	Taqman	PB	751	334	330	87	1243	532	550	161	0.315
Catucci	2010	Germany	Caucasian	Taqman	PB	1101	432	512	157	1496	584	696	216	0.711
Hu	2009	China	Asian	PCR-RFLP	PB	1009	239	483	287	1093	218	517	358	0.207
Hoffman	2009	USA	Caucasian	MassARRAY	HB	426	181	209	36	466	166	229	71	0.583
**miR499 (rs3746444 A > G)**						**Total**	**AA**	**AG**	**GG**	**Total**	**AA**	**AG**	**GG**	
Dai	2016	China	Asian	MassARRAY	HB	560	407	135	18	583	463	109	11	0.130
Qi	2015	China	Asian	TaqMan	HB	321	152	117	52	290	141	112	37	0.053
He	2015	China	Asian	MassARRAY	HB	450	184	177	89	450	203	188	59	0.143
Alshatwi	2012	Arabia	Caucasian	TaqMan	HB	100	30	62	8	100	45	40	15	0.227
Catucci	2010	Italy	Caucasian	Taqman	PB	756	414	295	47	1242	704	452	86	0.250
Catucci	2010	Germany	Caucasian	Taqman	PB	823	536	250	37	925	601	290	34	0.893
Hu	2009	China	Asian	PCR-RFLP	PB	1009	707	258	44	1093	816	248	29	0.057

Notes: PB: Population-based; HB: Hospital-based.

### Association between miRNA-146a rs2910164 polymorphism and BC susceptibility

**Figure 3:**
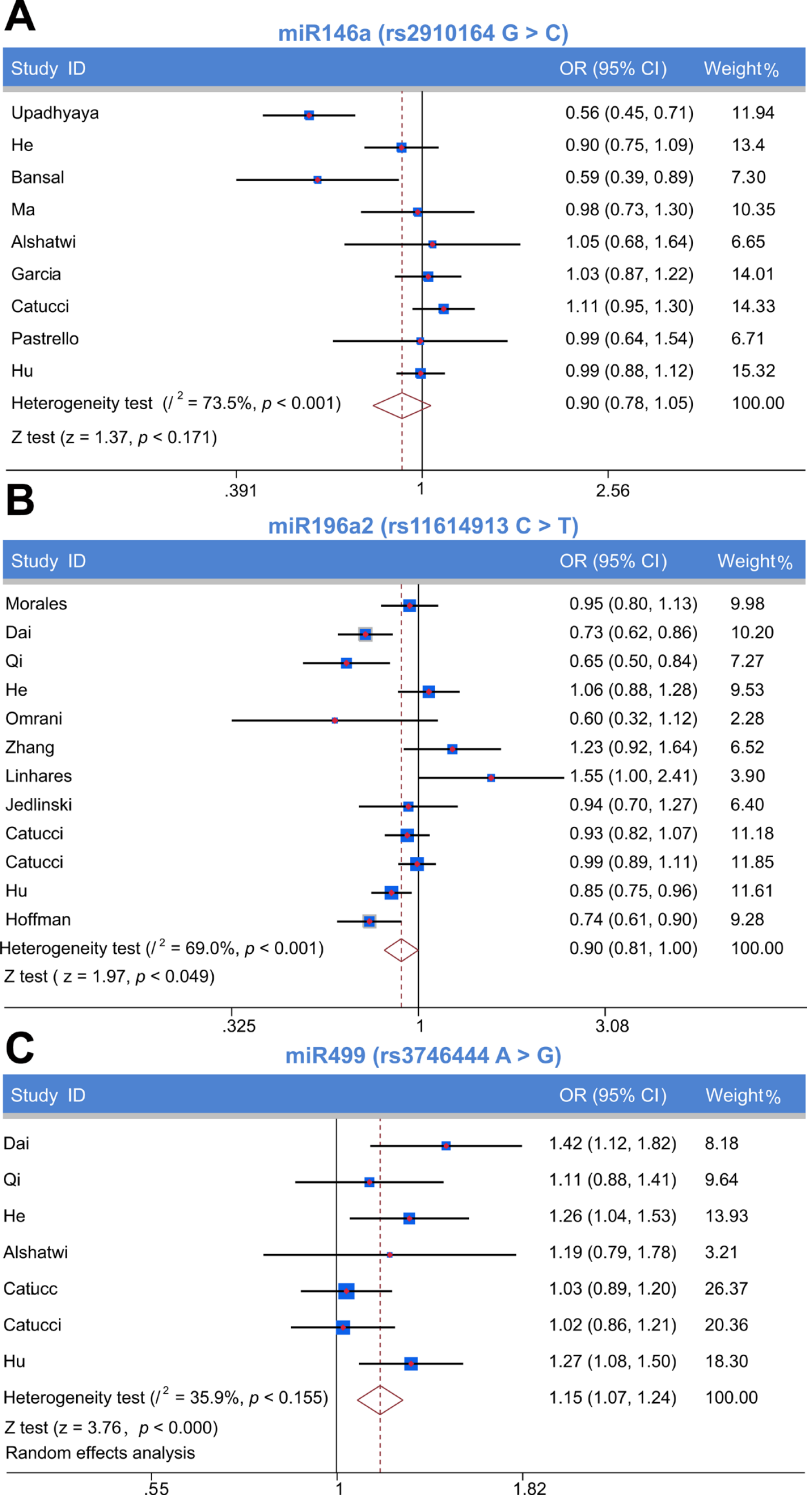
Meta-analysis of the relationship between miR-146a rs2910164, miR-196a2 rs11614913, and miR-499 rs3746444 polymorphisms and breast cancer risk Forest plot of allelic contrast model (**A**) miR-146a rs2910164, (**B**) miR-196a2 rs11614913, (**C**) miR-499 rs3746444).

We firstly assessed the association between miRNA-146a rs2910164 polymorphism and BC susceptibility. Significant heterogeneity was identified by *Q*-test and *I^2^* statistic under all genetic models except the heterozygote. Therefore, except for the latter, the random-effects model was used for all models. No significant associations were identified for any genetic model (C vs. G: OR = 0.90, 95% CI: 0.78–1.05; CC vs. GG: OR = 0.86, 95% CI: 0.62–1.20; GC vs. GG: OR = 0.95, 95% CI: 0.86–1.05; CC + GC vs. GG: OR = 0.89, 95% CI: 0.75–1.07; CC vs. GG + GC: OR = 0.89, 95% CI: 0.69–1.16) (Figure 3A, Table 2).

**Table 2:** Meta-analysis of the relationships between miR146a (rs2910164 G > C) polymorphism and breast cancer risk

Group	Included studies	Genotype models	OR (95% CI)	*Z*	*P*	Heterogeneity test	
						*P_h_*	*I^2^*
Total	9 studies	C vs. G	0.90 (0.78, 1.05)	1.37	0.171	0	73.50%
		CC vs. GG	0.86 (0.62, 1.20)	0.89	0.374	0.001	68.90%
		GC vs. GG	0.95 (0.86, 1.05)	0.98	0.326	0.061	46.40%
		CCcGC vs. GG	0.89 (0.75, 1.07)	1.26	0.209	0.005	63.40%
		CC vs. GG + GC	0.89 (0.69, 1.16)	0.88	0.379	0.005	63.90%
Asian	4 studies	C vs. G	0.91 (0.78, 1.06)	1.24	0.213	0.112	49.90%
		CC vs. GG	0.93 (0.76, 1.13)	0.73	0.463	0.701	0.00%
		GC vs. GG	0.88 (0.66, 1.19)	0.83	0.407	0.08	55.60%
		CC + GC vs. GG	0.86 (0.65, 1.14)	1.05	0.296	0.083	55.00%
		CC vs. GG + GC	0.93 (0.80, 1.07)	1.05	0.293	0.693	0.00%
Caucasian	5 studies	C vs. G	0.92 (0.70, 1.20)	0.62	0.533	0	83.40%
		CC vs. GG	0.85 (0.41, 1.80)	0.42	0.677	0	83.50%
		GC vs. GG	0.93 (0.76, 1.14)	0.66	0.506	0.088	50.60%
		CC + GC vs. GG	0.91 (0.70, 1.19)	0.68	0.494	0.005	73.30%
		CC vs. GG + GC	0.88 (0.45, 1.74)	0.36	0.72	0	80.70%
Population-based	6 studies	C vs. G	0.87 (0.71, 1.07)	1.3	0.193	0	83.10%
		CC vs. GG	0.85 (0.52, 1.38)	0.66	0.507	0	80.30%
		GC vs. GG	0.94 (0.84, 1.04)	1.19	0.236	0.015	64.70%
		CC + GC vs. GG	0.85 (0.67, 1.08)	1.37	0.172	0.001	76.60%
		CC vs. GG + GC	0.89 (0.59, 1.36)	0.53	0.599	0.001	76.30%
Hospital-based	3 studies	C vs. G	0.94 (0.81, 1.09)	0.87	0.383	0.771	0.00%
		CC vs. GG	0.87 (0.63, 1.20)	0.87	0.383	0.91	0.00%
		GC vs. GG	1.05 (0.80, 1.37)	0.33	0.739	0.916	0.00%
		CC + GC vs. GG	0.99 (0.77, 1.29)	0.05	0.957	0.877	0.00%
		CC vs. GG + GC	0.85 (0.68, 1.08)	1.32	0.187	0.769	0.00%
Sample-size < 500	4 studies	C vs. G	0.88 (0.69, 1.14)	0.96	0.34	0.167	40.70%
		CC vs. GG	0.91 (0.57, 1.45)	0.41	0.684	0	83.50%
		GC vs. GG	0.81 (0.62, 1.06)	1.53	0.127	0.133	46.50%
		CC + GC vs. GG	0.83 (0.58, 1.19)	1.01	0.313	0.123	48.10%
		CC vs. GG + GC	0.96 (0.66, 1.39)	0.22	0.827	0.797	0.00%
Sample size ≥ 500	5 studies	C vs. G	0.91 (0.76, 1.10)	0.99	0.321	0	83.80%
		CC vs. GG	0.85 (0.54, 1.32)	0.74	0.46	0.701	0.00%
		GC vs. GG	0.98 (0.87, 1.09)	0.43	0.667	0.097	49.00%
		CC + GC vs. GG	0.92 (0.74, 1.14)	0.77	0.441	0.005	73.00%

Notes: OR = Odds ratios; 95% CI = 95% confidence intervals.

Next, subgroup analysis was carried out according to race. No significant association was found in any genetic model for Asians and Caucasians. Subgroup analysis based on the source of controls revealed no significant association between any genetic model and either population-based or hospital-based groups; also, no associations were detected for sample sizes < 500 and ≥ 500 (Table 2).

### Association between miRNA-196a2 rs11614913 polymorphism and BC susceptibility

The association between miR-196a2 rs11614913 polymorphism and the risk of BC was tested using the random-effects model, due to the presence of significant heterogeneity, for the allelic contrast model and the homozygote, dominant, and recessive models, while the fixed-effects model was used for the heterozygote model. A significantly decreased risk of BC was observed under the allelic contrast model and the recessive model (T vs. C, OR = 0.90, 95% CI: 0.81–1.00; TT vs. CC + TC, OR = 0.86, 95% CI: 0.74–1.00; Table 3; Figure 3B).

**Table 3:** Meta-analysis of the relationships between miR196a2 (rs11614913 C > T) polymorphism and breast cancer risk

Group	Included studies	Genotype models	OR (95% CI)	*Z*	*P*	Heterogeneity test	
						*P_h_*	*I^2^*
Total	12 studies	**T vs. C**	**0.90 (0.81, 1.00)**	**1.97**	**0.049**	**0**	**69.00%**
		TT vs. CC	0.83 (0.67, 1.02)	1.76	0.079	0.001	68.00%
		TC vs. CC	0.92 (0.85, 1.00)	1.88	0.06	0.28	16.70%
		TT + TC vs. CC	0.88 (0.78, 1.01)	1.88	0.06	0.015	53.10%
		**TT vs. CC + TC**	**0.86 (0.74, 1.00)**	**2.01**	**0.044**	**0.008**	**58.00%**
Asian	6 studies	T vs. C	0.85 (0.71, 1.02)	1.75	0.08	0.001	75.50%
		TT vs. CC	0.78 (0.54, 1.12)	1.36	0.175	0.003	74.70%
		**TC vs. CC**	**0.85 (0.74, 0.99)**	**2.17**	**0.03**	**0.135**	**40.60%**
		TT + TC vs. CC	0.81 (0.63, 1.05)	1.59	0.112	0.021	62.40%
		TT vs. CC + TC	0.83 (0.67, 1.04)	1.65	0.099	0.014	67.90%
Caucasian	4 studies	T vs. C	0.91 (0.80, 1.03)	1.55	0.121	0.097	52.50%
		TT vs. CC	0.79 (0.59, 1.08)	1.49	0.136	0.041	63.70%
		TC vs. CC	0.95 (0.85, 1.06)	0.92	0.358	0.771	0.00%
		TT + TC vs. CC	0.94 (0.85, 1.05)	1.08	0.282	0.24	28.70%
		TT vs. CC + TC	0.83 (0.64, 1.08)	1.38	0.167	0.063	58.80%
Mix	2 studies	T vs. C	1.16 (0.72, 1.86)	0.62	0.538	0.042	75.90%
		TT vs. CC	1.31 (0.53, 3.28)	0.58	0.559	0.049	74.20%
		TC vs. CC	1.02 (0.80, 1.29)	0.15	0.877	0.196	40.10%
		TT + TC vs. CC	1.23 (0.63, 2.39)	0.6	0.548	0.084	66.60%
		TT vs. CC + TC	1.12 (0.65, 1.94)	0.41	0.678	0.129	56.60%
Population-based	6 studies	T vs. C	0.94 (0.85, 1.04)	1.21	0.228	0.126	41.90%
		TT vs. CC	0.89 (0.74, 1.07)	1.28	0.2	0.211	31.60%
		TC vs. CC	0.94 (0.84, 1.04)	1.22	0.224	0.476	0.00%
		TT + TC vs. CC	0.91 (0.81, 1.03)	1.44	0.151	0.263	22.70%
		TT vs. CC + TC	0.92 (0.81, 1.05)	1.23	0.22	0.339	11.70%
Hospital-based	6 studies	T vs. C	0.87 (0.72, 1.05)	1.41	0.159	0	78.90%
		TT vs. CC	0.76 (0.51, 1.13)	1.35	0.178	0	78.30%
		TC vs. CC	0.90 (0.79, 1.03)	1.5	0.132	0.132	41.00%
		TT + TC vs. CC	0.87 (0.68, 1.11)	1.11	0.266	0.007	68.60%
		TT vs. CC + TC	0.79 (0.60, 1.02)	1.79	0.073	0.007	68.60%
Sample-size < 500	4 studies	T vs. C	1.07 (0.79, 1.45)	0.43	0.667	0.058	59.90%
		TT vs. CC	1.43 (0.81, 2.52)	1.23	0.218	0.178	42.10%
		TC vs. CC	0.96 (0.70, 1.31)	0.25	0.801	0.149	43.80%
		TT + TC vs. CC	1.07 (0.66, 1.76)	0.28	0.778	0.066	58.30%
		TT vs. CC + TC	1.21 (0.92, 1.59)	1.36	0.173	0.502	0.00%
Sample size ≥ 500	8 studies	**T vs. C**	**0.87 (0.78, 0.96)**	**2.74**	**0.006**	**0.002**	**70.00%**
		**TT vs. CC**	**0.75 (0.61, 0.93)**	**2.66**	**0.008**	**0.003**	**68.00%**
		TC vs. CC	0.92 (0.85, 1.00)	1.88	0.06	0.349	10.50%
		**TT + TC vs. CC**	**0.86 (0.76, 0.98)**	**2.22**	**0.026**	**0.029**	**55.20%**
		**TT vs. CC + TC**	**0.80 (0.69, 0.93)**	**2.94**	**0.003**	**0.03**	**54.90%**

Notes: OR = Odds ratios; 95% CI = 95% confidence intervals.

In subgroup analysis by race, a significantly decreased risk of BC was observed for Asians under the heterozygote model (TC vs. CC: OR = 0.85, 95% CI: 0.74–0.99). In Caucasians, in contrast, no association was detected between miR-196a2 rs11614913 and BC risk for any genotype model. Similarly, no relationship was found for any genotype model in the mixed-race subgroup. Results of subgroup analysis based on the source of controls showed no significant association between any genetic model and either population-based or hospital-based controls. We also found no significant association for sample size < 500 under any genetic model, although a sample size ≥ 500 was associated with decreased BC risk in all models except the heterozygote (T vs. C: OR = 0.87, 95% CI: 0.78–0.96; TT vs. CC: OR = 0.75, 95% CI: 0.61–0.93; TT + TC vs. CC: OR = 0.86, 95% CI: 0.76–0.98; TT vs. CC + TC: OR = 0.80, 95% CI: 0.69–0.93; Table 3).

### Association between miRNA-499 rs3746444 polymorphism and BC susceptibility

The association between miR-499 rs3746444 polymorphism and the risk of BC was examined by applying the fixed-effects model to assess all genetic models. A significantly increased risk of BC was observed for all genetic models (G vs. A: OR = 1.15, 95% CI: 1.07–1.24; GG vs. AA: OR = 1.32, 95% CI: 1.10–1.58; GA vs. AA: OR = 1.13, 95% CI: 1.03–1.24; GG + GA vs. AA: OR = 1.16, 95% CI: 1.06–1.27; GG vs. AA + GA: OR = 1.27, 95% CI: 1.06–1.51; Figure 3C).

Subgroup analysis was performed for the Asian population, where a positive association was identified under all genetic models (G vs. A: OR = 1.26, 95% CI: 1.14–1.40; GG vs. AA: OR = 1.60, 95% CI: 1.26–2.04; GA vs. AA: OR = 1.17, 95% CI: 1.02–1.33; GG + GA vs. AA: OR = 1.25, 95% CI: 1.10–1.41; GG vs. AA + GA: OR = 1.60, 95% CI: 1.27–2.02). In contrast, no association was found for Caucasians under any genetic model. Subgroup analysis by source of controls indicated an association with hospital-based controls under all genetic models (G vs. A: OR = 1.25, 95% CI: 1.11–1.41; GG vs. AA: OR = 1.48, 95% CI: 1.13–1.93; GA vs. AA: OR = 1.21, 95% CI: 1.02–1.43; GG + GA vs. AA: OR = 1.28, 95% CI: 1.09–1.49; GG vs. AA + GA: OR = 1.39, 95% CI: 1.08–1.79). Also, a significant association was identified between all genetic models and sample size ≥ 500 (G vs. A: OR = 1.15, 95% CI: 1.07–1.24; GG vs. AA: OR = 1.34, 95% CI: 1.12–1.62; GA vs. AA: OR = 1.11, 95% CI: 1.01–1.22; GG + GA vs. AA: OR = 1.15, 95% CI: 1.05–1.25; GG vs. AA + GA: OR = 1.32, 95% CI: 1.10–1.58), and between the dominant and heterozygote models and sample size < 500 (GA vs. AA: OR = 2.33, 95% CI: 1.26–4.28; GG + GA vs. AA: OR = 1.91, 95% CI: 1.07–3.41; Table 4).

**Table 4:** Meta-analysis of the relationships between miR499 (rs3746444 A > G) polymorphism and breast cancer risk

Group	Included studies	Genotype models	OR (95% CI)	*Z*	*P*	Heterogeneity test	
						*P_h_*	*I^2^*
Total	7 studies	**G vs. A**	**1.15 (1.07, 1.24)**	**3.76**	**0**	**0.155**	**35.90%**
		**GG vs. AA**	**1.32 (1.10, 1.58)**	**2.99**	**0.003**	**0.239**	**24.80%**
		**GA vs. AA**	**1.13 (1.03, 1.24)**	**2.48**	**0.013**	**0.078**	**47.20%**
		**GG + GA vs. AA**	**1.16 (1.06, 1.27)**	**3.23**	**0.001**	**0.151**	**36.40%**
		**GG vs. AA + GA**	**1.27 (1.06, 1.51)**	**2.65**	**0.008**	**0.071**	**48.30%**
Asian	4 studies	**G vs. A**	**1.26 (1.14, 1.40)**	**4.52**	**0**	**0.567**	**0.00%**
		**GG vs. AA**	**1.60 (1.26, 2.04)**	**3.84**	**0**	**0.795**	**0.00%**
		**GA vs. AA**	**1.17 (1.02, 1.33)**	**2.31**	**0.021**	**0.321**	**14.20%**
		**GG + GA vs. AA**	**1.25 (1.10, 1.41)**	**3.46**	**0.001**	**0.492**	**0.00%**
		**GG vs. AA + GA**	**1.60 (1.27, 2.02)**	**4.01**	**0**	**0.77**	**0.00%**
Caucasian	3 studies	G vs. A	1.04 (0.93, 1.15)	0.69	0.493	0.794	0.00%
		GG vs. AA	1.01 (0.76, 1.34)	0.06	0.955	0.605	0.00%
		GA vs. AA	1.19 (0.88, 1.60)	1.1	0.271	0.026	72.50%
		GG + GA vs. AA	1.14 (0.89, 1.39)	0.96	0.335	0.113	54.20%
		GG vs. AA + GA	0.94 (0.71, 1.24)	0.45	0.65	0.194	39.10%
Population-based	3 studies	G vs. A	1.10 (1.00, 1.20)	1.96	0.05	0.114	53.90%
		GG vs. AA	1.19 (0.93, 1.53)	1.4	0.162	0.124	52.00%
		GA vs. AA	1.09 (0.97, 1.22)	1.48	0.139	0.327	10.60%
		GG + GA vs. AA	1.11 (0.99, 1.23)	1.81	0.07	0.229	32.10%
		GG vs. AA + GA	1.16 (0.90, 1.48)	1.16	0.247	0.116	53.50%
Hospital-based	4 studies	**G vs. A**	**1.25 (1.11, 1.41)**	**3.63**	**0**	**0.554**	**0.00%**
		**GG vs. AA**	**1.48 (1.13, 1.93)**	**2.87**	**0.004**	**0.474**	**0.00%**
		**GA vs. AA**	**1.21 (1.02, 1.43)**	**2.22**	**0.027**	**0.042**	**63.30%**
		**GG + GA vs. AA**	**1.28 (1.09, 1.49)**	**3.03**	**0.002**	**0.223**	**31.50%**
		**GG vs. AA + GA**	**1.39 (1.08, 1.79)**	**2.6**	**0.009**	**0.104**	**51.30%**
Sample-size < 500	1 study	G vs. A	1.19 (0.79, 1.78)	0.83	0.408	NA	NA
		GG vs. AA	0.80 (0.30, 2.12)	0.45	0.654	NA	NA
		**GA vs. AA**	**2.33 (1.26, 4.28)**	**2.71**	**0.007**	**NA**	**NA**
		**GG + GA vs. AA**	**1.91 (1.07, 3.41)**	**2.18**	**0.029**	**NA**	**NA**
		GG vs. AA + GA	0.49 (0.20, 1.22)	1.53	0.126	NA	NA
Sample size ≥ 500	6 studies	**G vs. A**	**1.15 (1.07, 1.24)**	**3.67**	**0**	**0.097**	**46.40%**
		**GG vs. AA**	**1.34 (1.12, 1.62)**	**3.13**	**0.002**	**0.226**	**27.90%**
		**GA vs. AA**	**1.11 (1.01, 1.22)**	**2.07**	**0.039**	**0.326**	**13.90%**
		**GG + GA vs. AA**	**1.15 (1.05, 1.25)**	**2.93**	**0.003**	**0.258**	**23.50%**
		**GG vs. AA + GA**	**1.32 (1.10, 1.58)**	**3.03**	**0.002**	**0.202**	**31.10%**

Notes: OR = Odds ratios; 95% CI = 95% confidence intervals; NA = Not available.

### Publication bias

**Figure 4:**
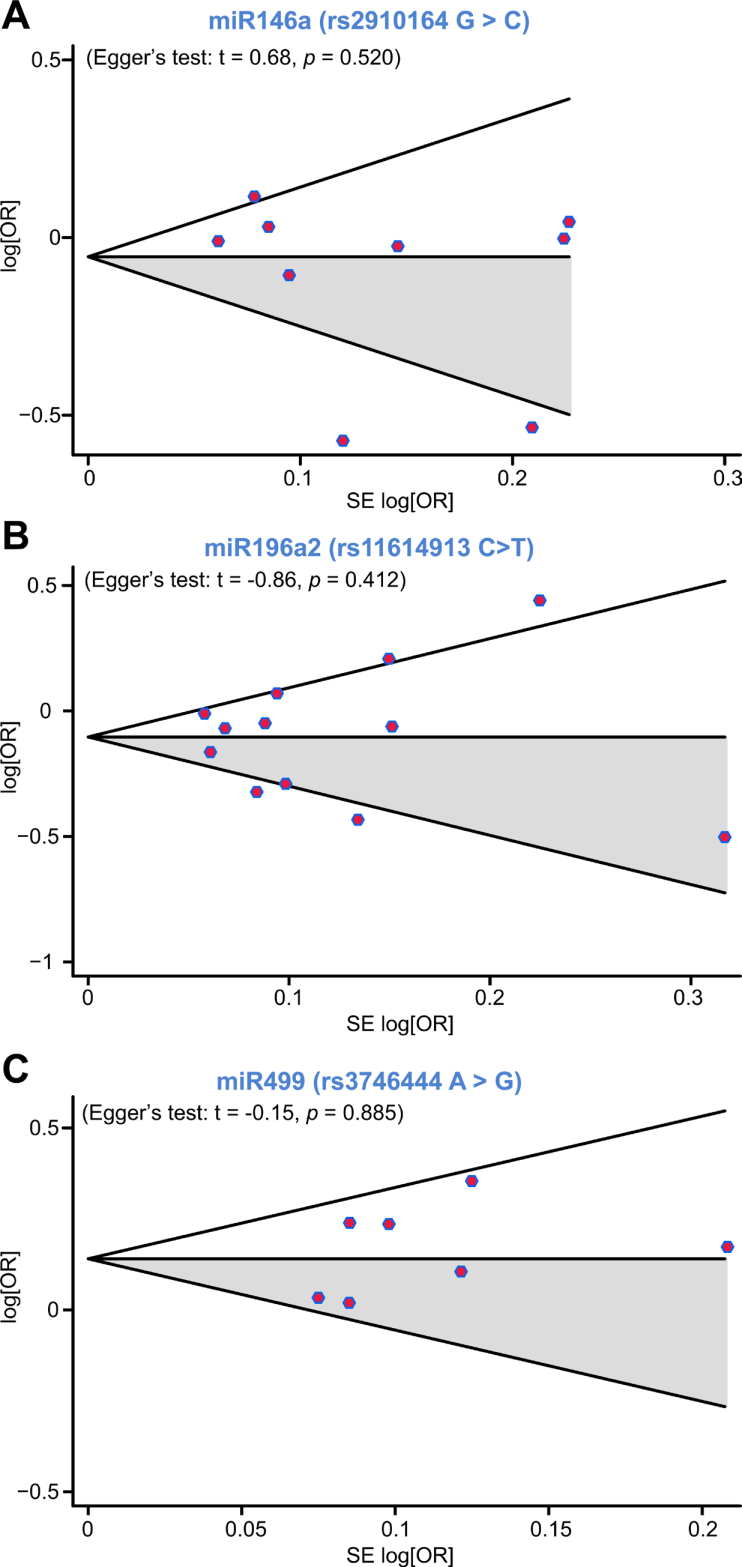
Begg's funnel plot for publication bias analysis under the allelic contrast model (**A**) miR-146a rs2910164, (**B**) miR-196a2 rs11614913, (**C**) miR-499 rs3746444).

We utilized Begg's funnel plot and Egger's test to evaluate publication bias. No evidence of publication bias was found for the association between miR-146a rs2910164, miR-196a2 rs11614913, and miR-499 rs3746444 polymorphisms and BC susceptibility using Begg's funnel for the allelic contrast model (Figure 4). Egger's test also suggested no publication bias for the homozygote (rs2910164: *P* = 0.607; rs11614913: *P* = 0.624; rs3746444: *P* = 0.975), heterozygote (rs2910164: *P* = 0.298; rs11614913: *P* = 0.948; rs3746444: *P* = 0.207), dominant (rs2910164: *P* = 0.286; rs11614913: *P* = 0.942; rs3746444: *P* = 0.166) and recessive (rs2910164: *P* = 0.724; rs11614913: *P* = 0.728; rs3746444: *P* = 0.653) models.

### Sensitivity analysis

**Figure 5:**
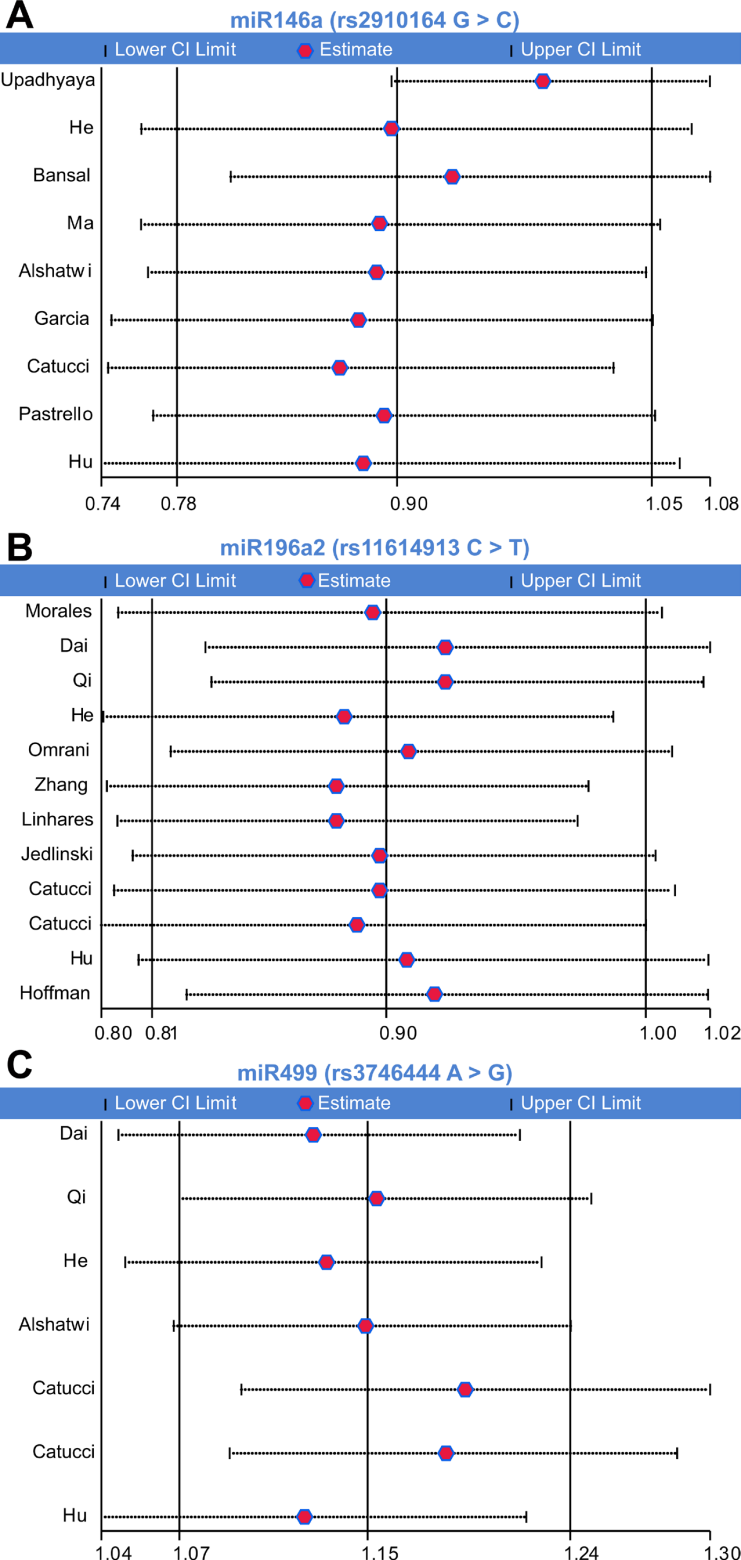
Influence of individual studies on the overall OR under the allelic contrast model (**A**) miR-146a rs2910164, (**B**) miR-196a2 rs11614913, (**C**) miR-499 rs3746444).

To examine the influence exerted by individual studies on the pooled ORs, sensitivity analysis in the allelic contrast model was performed by successively deleting each participant study. We confirmed that the omission of any single study did not significantly affect the overall results (Figure 5).

## DISCUSSION

MiRNAs mediate degradation or translational repression by binding to the 3′UTR and 5′UTR of the target mRNA [2]. SNPs, the most common source of genetic sequence variation, can affect the function of miRNAs by altering primary transcript formation, pre-miRNA maturation, or miRNA-mRNA interactions [11, 37]. Even minor variations in miRNAs could have an enormous effect on the expression of different target genes and thus lead to susceptibility to several diseases including BC [38]. Murria Estal et al. identified miRNA profiles related to breast cancer features like node involvement, histological grade, ER, PR, and HER2 expression [39]. SNPs in miR-146a (rs2910164), miR-196a2 (rs11614913), and miR-499 (rs3746444) have been suggested to be predictive biomarkers for patients with BC [12, 14, 15, 26–28, 30, 31, 35, 40], although the studies in question provided inconsistent results. This lack of consensus prompted us to perform a comprehensive meta-analysis on the association of these three miRNAs polymorphisms and BC risk.

The miR-146a human gene is located on chromosome 5 at locus 5q34 and has been linked with BRCA1/BRCA2 activity. The SNP rs2910164 is located in the middle of the miRNA stem hairpin and leads to a change from a G:U pair to a C:U mismatch in the stem structure of the precursor molecule, altering the expression of mature miR-146a [41]. This SNP has been associated with the risk of various cancers, among them hepatocellular and bladder carcinomas [41, 42], evidencing also cancer-specific and ethnicity-dependent effects [43, 44]. Our analysis of miR-146a rs2910164, which included 4,441 cases and 3,899 controls from nine studies, revealed however no association with BC in both overall comparison and subgroup analysis by race, source of control, and sample size. In accordance with this conclusion, studies by Catucci et al. [14] and Alshatwi et al. [24] also failed to demonstrate a link between rs2910164 and BC risk.

The rs11614913 polymorphism in miR-196a2, located in the mature sequence of miR-196a-3P, may affect pre-miRNA maturation and confer diminished capacity to regulate target genes [11, 27]. Epidemiology studies have also revealed an association between rs11614913 and risk for multiple cancers; however, results were conflicting [42, 45]. Similarly, Linhares et al. [32] found that individuals carrying the CC genotype of rs11614913 in miR-196a2 had decreased BC risk, whereas Gao et al. [46] found instead a positive association between this genotype and BC risk. On the other hand, Dai et al. concluded that rs11614913 may reduce the risk of BC under the recessive model [47]. However, our meta-analysis involving twelve studies with 5,792 cases and 7,159 controls demonstrated an association of rs11614913 with decreased risk of BC both in the allelic contrast model and the recessive model. In subgroup analysis, a significant association was observed between rs11614913 and reduced risk of BC for the heterozygote model in Asians, and for all, except the heterozygote, genetic models when sample size ≥ 500. The discrepancies may derive from different sample sizes, races, and genetic backgrounds of the studies’ groups.

Rs3746444, located at the 3p region of mature miR-499, involves a A:U to G:U mismatch in the stem structure of the precursor molecule, leading to altered processing and expression of the mature transcript [48]. The presence of this mismatch would affect Sox6 and Rod1 genes, which are important in the etiology of several cancers [49]. A number of studies investigating the association between rs3746444 and cancer risk have found that this SNP has distinct effects on different populations and cancer types. Dai et al. found that rs3746444 may be related to increased risk of BC under the allelic contrast, homozygote, and recessive models [47]. Our meta-analysis, assessing seven studies with 4,019 cases and 4,683 controls, showed that carriers of the rs3746444 GG genotype and GG + GA genotypes are at a significantly increased risk of developing BC when compared with those carrying the AA genotype. Also, Asians and hospital-based control subgroups demonstrated a significant association with increased risk of BC under all genetic models, but no significant association was found for Caucasians and for population-based source of control under any model. Thus, our results suggest oncogenic mechanisms are distinctly influenced by specific genetic backgrounds across populations.

Although the studies included in our meta-analysis differed from one another in numerous aspects, sensitivity analysis of miR-146a rs2710164, miR-196a2 rs11614913, and miR-499 rs3746444 indicated that the associations detected were not driven by any single one. Moreover, no publication bias was identified with either Begg's funnel plot or Egger's regression test. Finally, no limitations were imposed on our literature search, thus selection bias was well controlled.

Nevertheless, some limitations in this meta-analysis are noteworthy. Firstly, the number of included studies for the miR-499 rs3746444 polymorphism was limited. Secondly, there exists a certain degree of heterogeneity in some genetic models of rs2710164, rs11614913 and rs3746444. After subgroup analysis stratified by race, it could be established that heterogeneity was significantly reduced for Asians in some genetic models of rs11614913 and in all the genetic models of rs3746444. Thus, it could be assumed that the observed heterogeneity resulted, at least in part, from racial differences, which may have impacted the results of our study.

In conclusion, our results indicated that the rs2910164 (G > C) polymorphism in miR-146a may not be associated with susceptibility to BC; the rs11614913 (C > T) polymorphism in miR-196a2 is significantly associated with decreased BC risk; and the rs3746444 (A > G) polymorphism in miR-499 is associated with increased BC risk, especially in Asians. Thus, rs11614913(C > T) and rs3746444 (A > G) appear to be both promising biomarkers to forecast BC risk and potential therapeutic targets. However, owing to the limitations mentioned above, these results should be treated with caution. To further verify and confirm these findings, well-designed, large scale case–control studies will be required.

## MATERIALS AND METHODS

### Search strategy and selection criteria

This meta-analysis was performed in accordance with the Preferred Reporting Items for Systematic Reviews and Meta-Analyses (PRISMA) statement [50]. To identify all published studies addressing the relationship between miRNA polymorphisms and BC risk, PubMed and Embase databases (last updated on July 20, 2016) were searched without publication type or date restrictions using the following keywords: breast cancer/carcinoma, miR-146a/rs2910164, miR-196a2/rs11614913, miR-499/rs3746444, and polymorphism/SNP/variation. The literature search was limited to English articles. We selected all potentially eligible studies for review.

### Study selection and data extraction

All the included studies were selected following the Strengthening the Reporting of Observational Studies in Epidemiology (STROBE) guidelines [51]. Eligible studies met the following criteria: (1) assessed the relationship between miR-146a/-196a2/-499 polymorphisms and BC risk; (2) had a case-control design; (3) addressed histologically confirmed BC; (4) had sufficient genotype data for further calculating odds ratios (ORs) and their 95% confidence intervals (95% CIs); (5) met Hardy-Weinberg equilibrium (HWE) in the control group (*P* > 0.05). Exclusion criteria included: (1) duplications of previous publications; (2) comments, meeting reports, reviews or editorials; (3) family-based studies of pedigrees; (4) studies with no detailed genotype data. When there were multiple publications from the same population, only the largest study was included. Study selection was done by two investigators independently, by screening the title, abstract and full-text. Any dispute was settled by discussion.

Data from eligible studies were extracted in duplicate by two investigators independently (Mu and Guo). Extracted data included author, year, country, race, genotyping method, source of control, genetic models of cases and controls, and *P* value for HWE (Table 1). These two authors checked the extracted data and approved it by consensus. If dissent existed, an additional investigator (Liu) would intervene to settle the disagreement. The quality of selected studies was assessed by two or more investigators independently, according to the Newcastle-Ottawa Scale (NOS) criteria [52]. As per the latter, studies must ascertain or include: cases with independent validation (NOS01); representativeness of the cases (NOS02); selection of controls from community controls (NOS03); controls with no history of disease (endpoint) (NOS04); appropriate study controls for the most important study factor (NOS05); study controls for any additional factor (NOS06); secure record (NOS07); structured interview where interviewer is blind to case/control status (NOS08); same method of ascertaining exposure for cases and controls (NOS09); same non-response rate for both groups (NOS10). The maximum NOS score is 10 points, and studies scoring 6 or higher were included in the meta-analysis.

### Statistical analysis

We calculated the *P* value of HWE in the control group using an online tool (http://ihg.gsf.de/cgi-bin/hw/hwa1.pl). The departure from HWE of SNP frequencies in the control group was assessed by *X^2^* test, and a *P* value < 0.05 was regarded as significant. Odds ratios (ORs) and 95% confidence intervals (CIs) were obtained to evaluate the strength of the association between miR-146a/-196a2/-499 SNPs and susceptibility to BC. Pooled ORs were determined for the allelic contrast model (miR-146a: C vs G, miR-196a2: T vs C, miR-499: G vs A), homozygote model (miR-146a: CC vs GG, miR-196a2: TT vs CC, miR-499: GG vs AA), heterozygote model (miR-146a: GC vs GG, miR-196a2: TC vs CC, miR-499: AG versus AA), dominant model (miR-146a: CC + GC vs GG, miR-196a2: TT + TC vs CC, GG + AG vs AA), and recessive model (miR-146a: CC vs CG + GG, miR-196a2: TT vs TC + CC, miR-499:GG vs AG + AA). The statistical significance of the pooled OR was evaluated by *Z* test and a *P* value of < 0.05 was regarded as significant. Inter-study heterogeneity was tested using a *X^2^*-based *Q*-test (with significance level *P* < 0.1) and *I^2^* statistic (with values greater than 50% indicating significant heterogeneity) [53]. According to the result of the heterogeneity test, the random model was chosen to assess OR and 95% CI when *P* < 0.05; conversely, the fixed model was selected when *P* > 0.05. Subgroup analysis was performed by race, source of control, and sample size. Sensitivity analysis was performed to evaluate the effect of each study on the combined ORs by omitting individual studies one at a time. Publication bias was checked by Begg's funnel plots [54] and Egger's regression test [55]. An asymmetric plot and a *P* < 0.05 for the Egger's test denoted a noteworthy publication bias. The trim-and-fill computation was used to estimate the effect of publication bias on the interpretation of the results [56]. Statistical analysis was conducted utilizing Stata12.0 Software.
